# Supplementary material for: A computational method for the systematic screening of reaction barriers in enzymes: searching for Bacillus circulans xylanase mutants with greater activity towards a synthetic substrate
Source: PeerJ. 2013 Jul 23;1:e111. doi: 10.7717/peerj.111 (PMC3728886; doi:10.7717/peerj.111)

Position 7

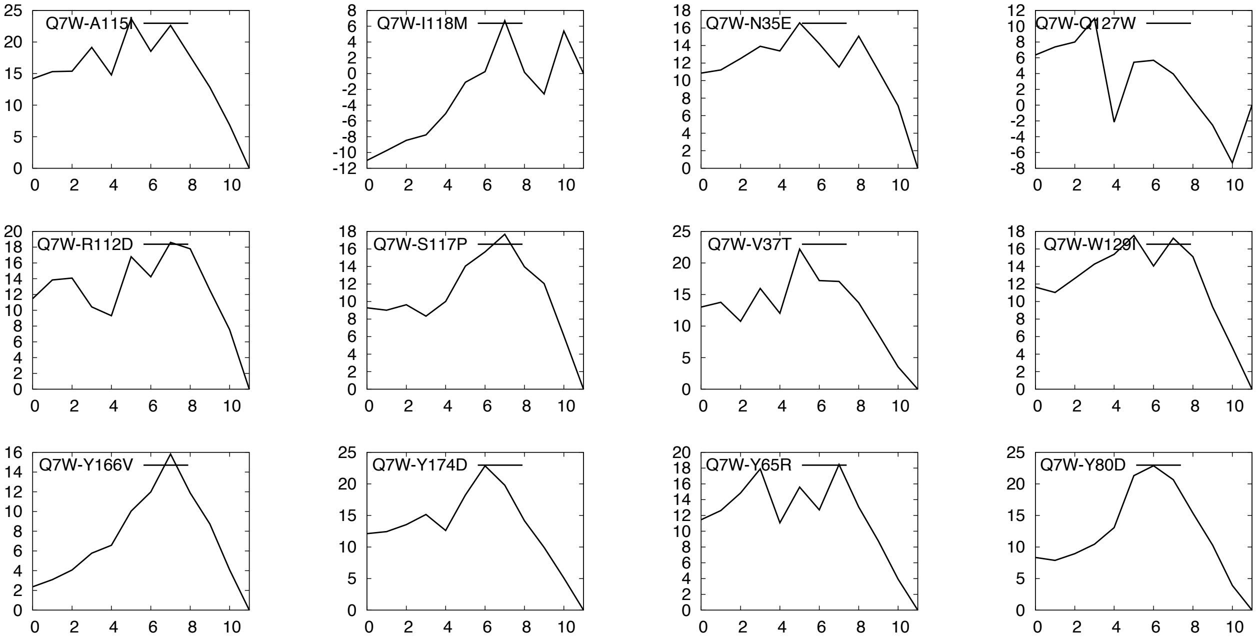
Q7W-A115I
Q7W-I118M
Q7W-N35E
Q7W-Q127W
Q7W-R112D
Q7W-S117P
Q7W-V37T
Q7W-W129I
Q7W-Y166V
Q7W-Y174D
Q7W-Y65R
Q7W-Y80D

Position 9

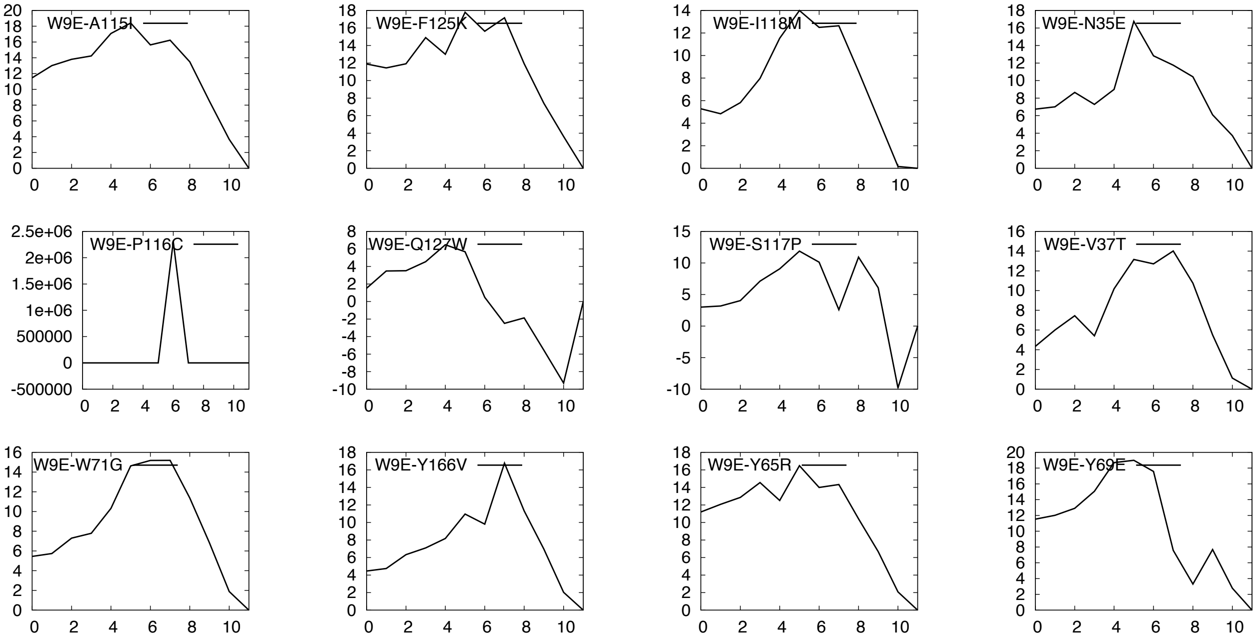

W9E-A115I
W9E-F125K
W9E-I118M
W9E-N35E
W9E-P116C
W9E-Q127W
W9E-S117P
W9E-V37T
W9E-W71G
W9E-Y166V
W9E-Y65R
W9E-Y69E

Position 35

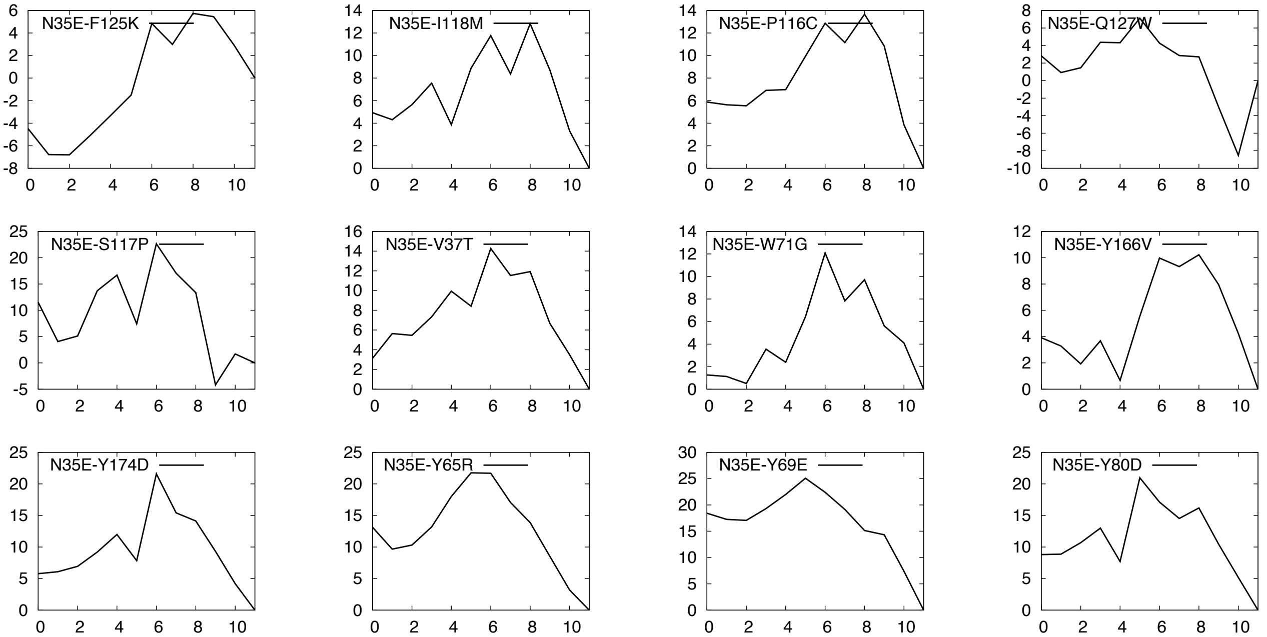

N35E-F125K
N35E-I118M
N35E-P116C
N35E-Q127W
N35E-S117P
N35E-V37T
N35E-W71G
N35E-Y166V
N35E-Y174D
N35E-Y65R
N35E-Y69E
N35E-Y80D

Position 37

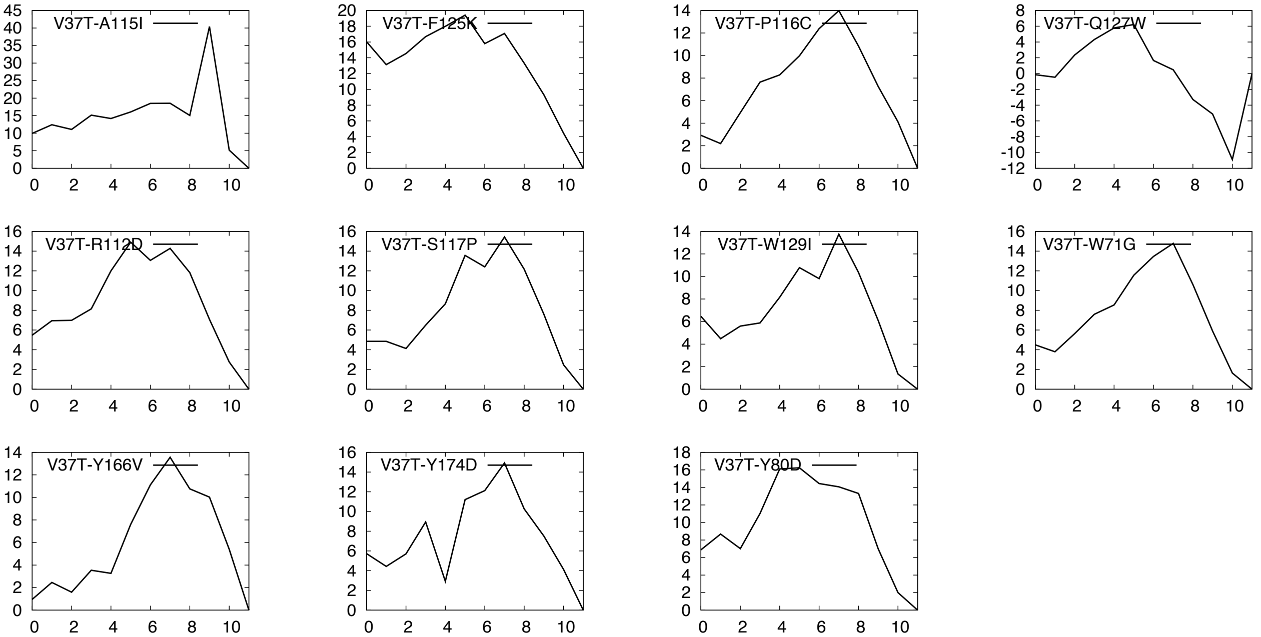
V37T-A115I
V37T-F125K
V37T-P116C
V37T-Q127W
V37T-R112D
V37T-S117P
V37T-W129I
V37T-W71G
V37T-Y166V
V37T-Y174D
V37T-Y80D

Position 65

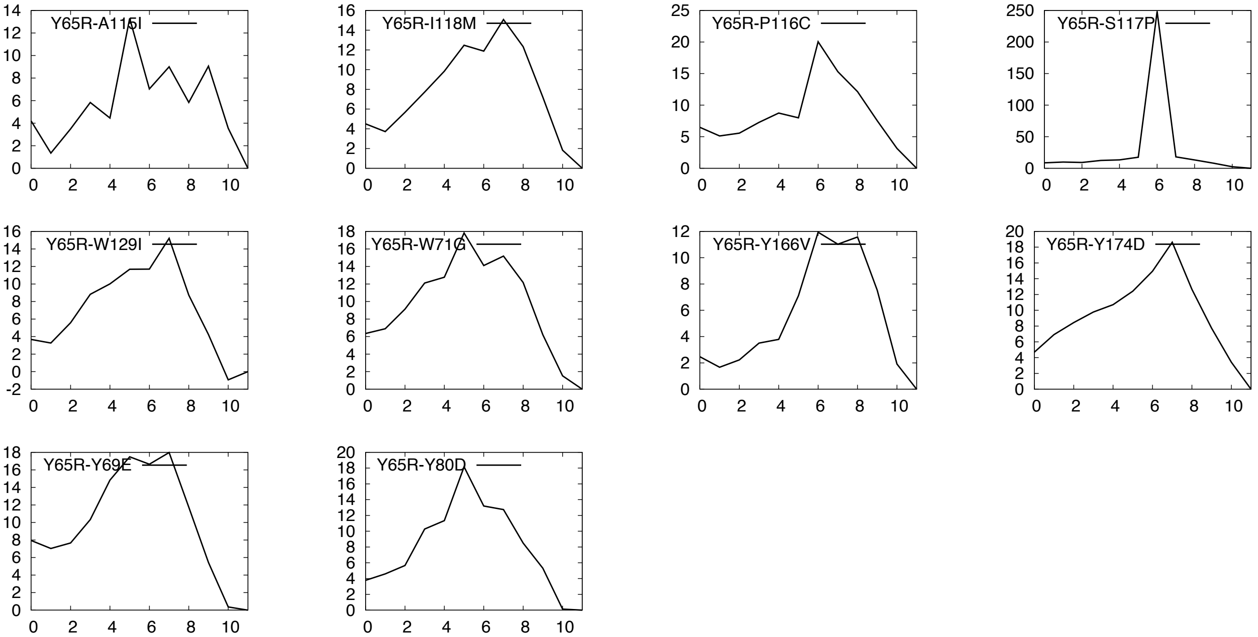
Y65R-A115I
Y65R-I118M
Y65R-P116C
Y65R-S117P
Y65R-W129I
Y65R-W71G
Y65R-Y166V
Y65R-Y174D
Y65R-Y69E
Y65R-Y80D

Position 69

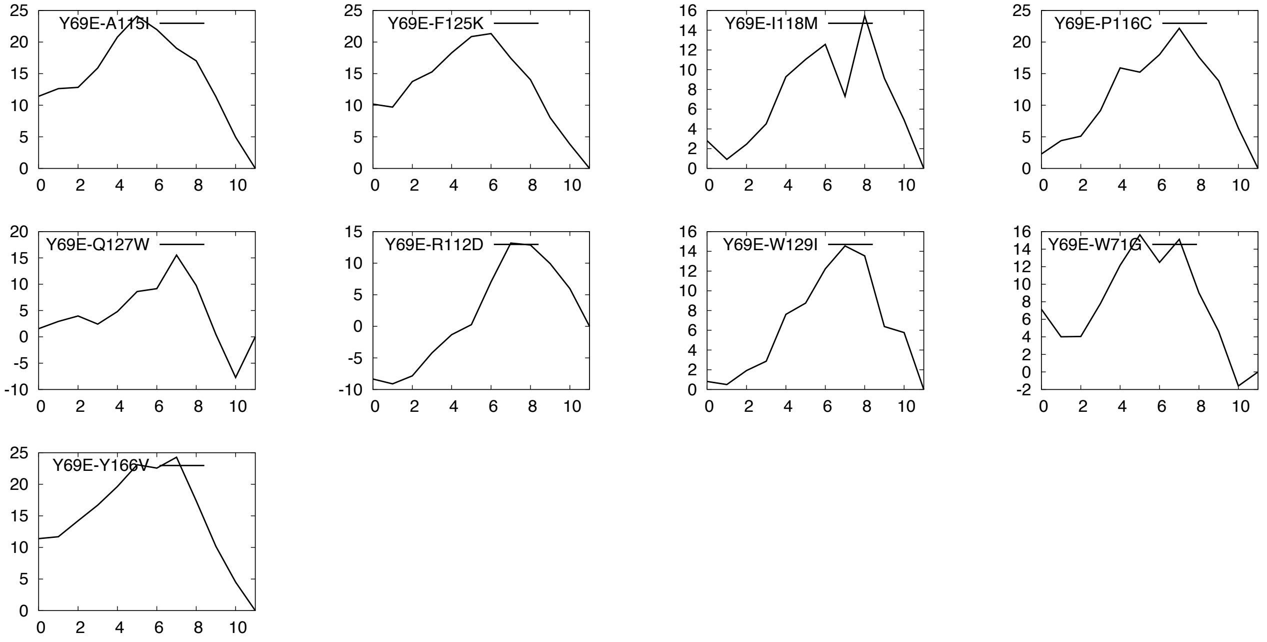

Y69E-A115I
Y69E-F125K
Y69E-I118M
Y69E-P116C
Y69E-Q127W
Y69E-R112D
Y69E-W129I
Y69E-W71G
Y69E-Y166V

Position 71

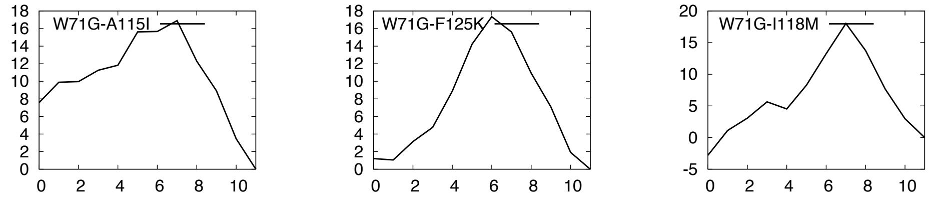

Position 80

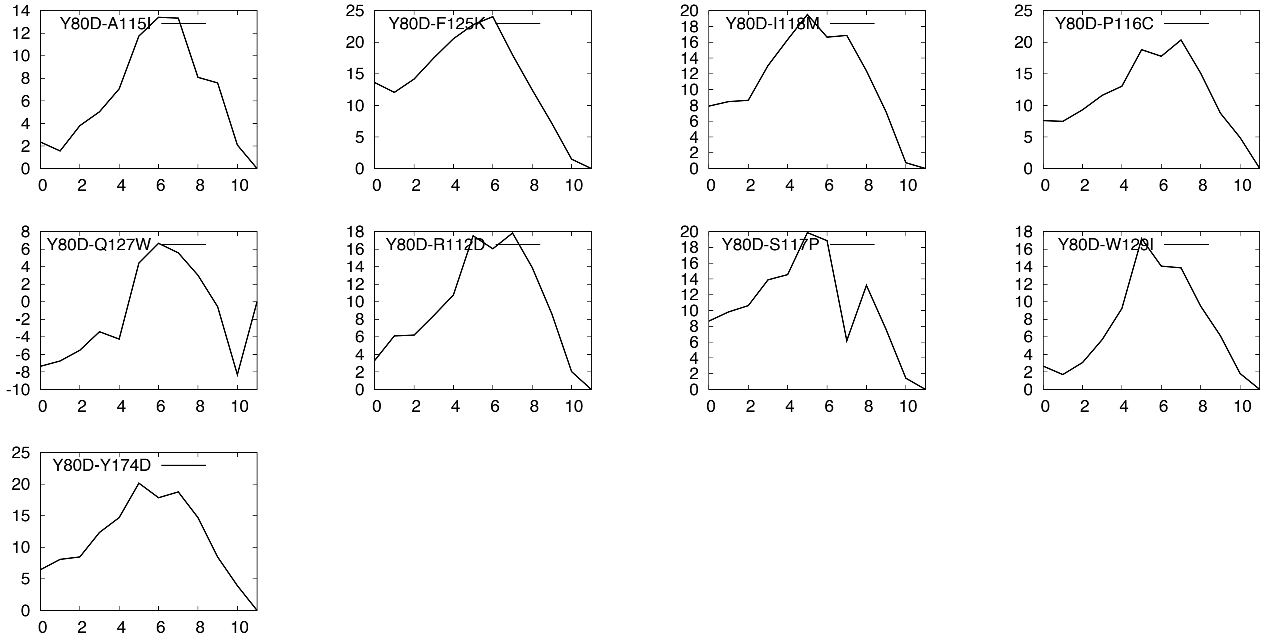

Y80D-A115I
Y80D-F125K
Y80D-I118M
Y80D-P116C
Y80D-Q127W
Y80D-R112D
Y80D-S117P
Y80D-W129I
Y80D-Y174D

Position 112

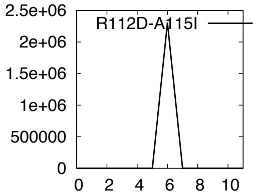

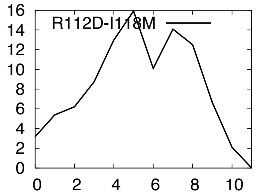

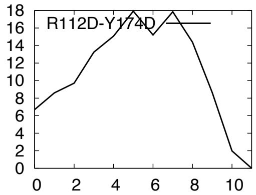

Position 115

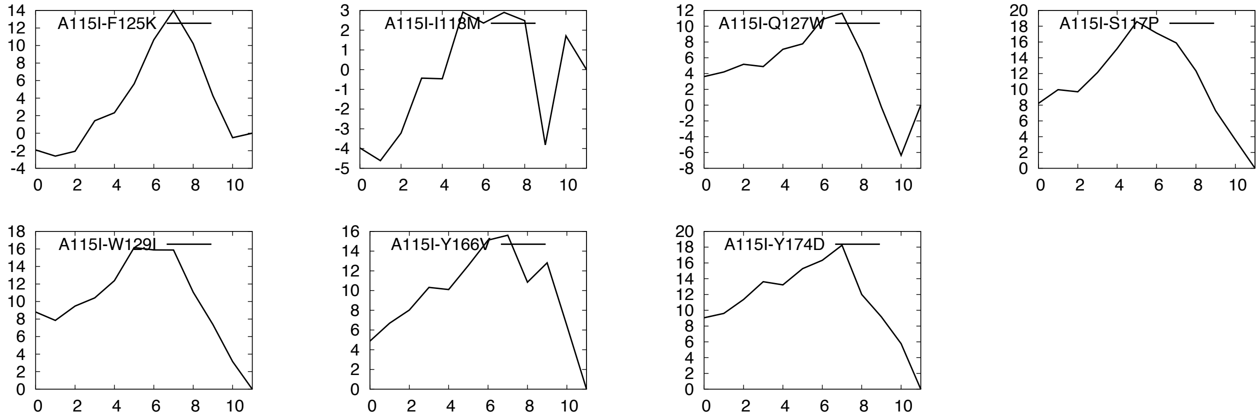

Position 116

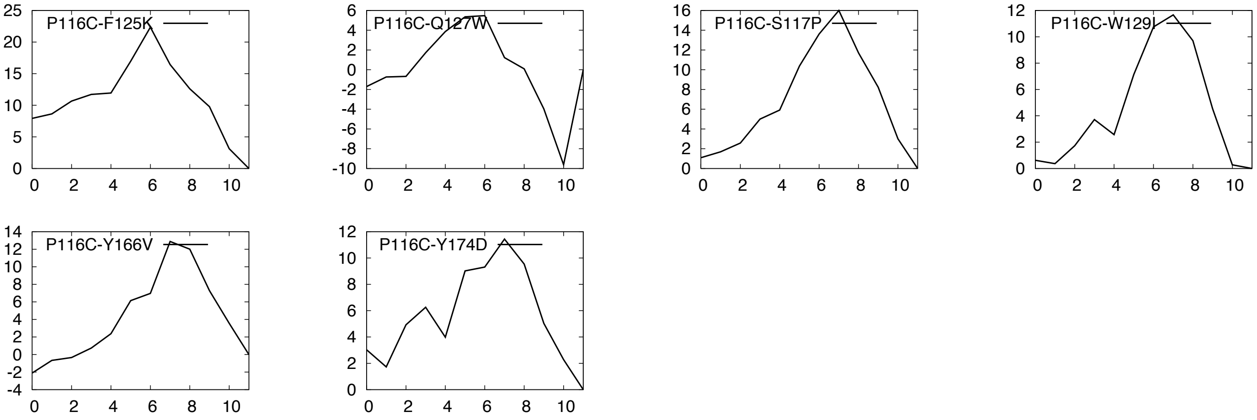

Position 117

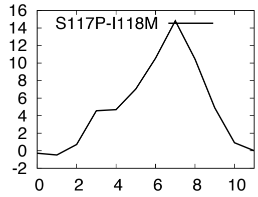

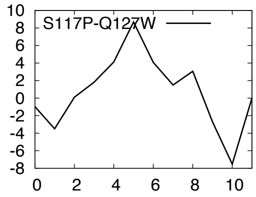

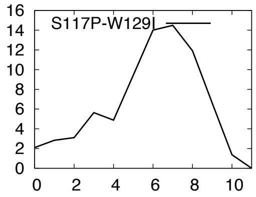

Position 118

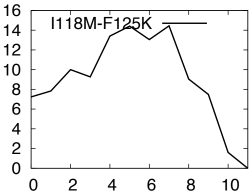

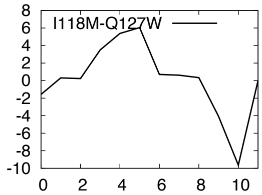

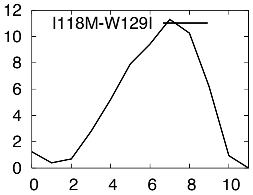

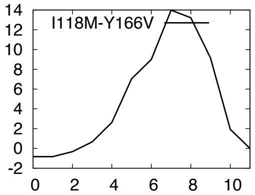

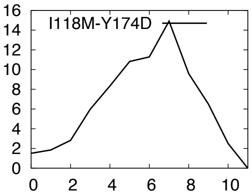

Position 125

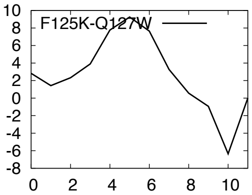

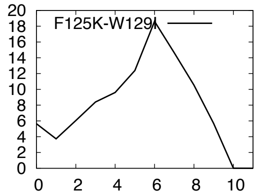

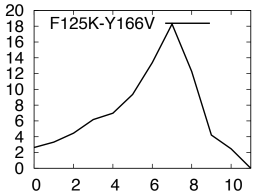

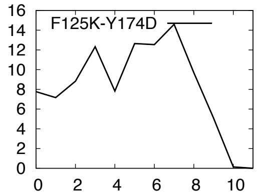

Position 127

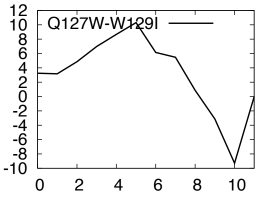

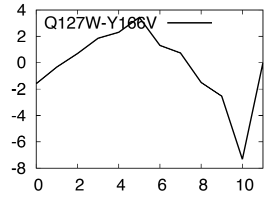

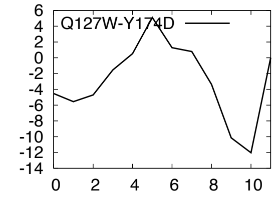

Position 129

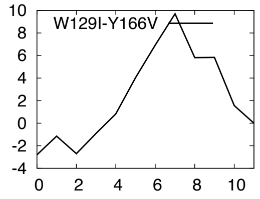

Position 166

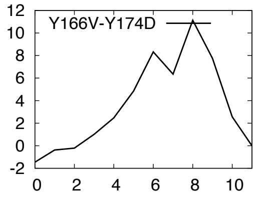

Supplement: Supplemental Information 1 [file peerj-01-111-s001.pdf]
