# Supplementary figures and images for: A computational method for the systematic screening of reaction barriers in enzymes: searching for Bacillus circulans xylanase mutants with greater activity towards a synthetic substrate
Source: PeerJ. 2013 Jul 23;1:e111. doi: 10.7717/peerj.111 (PMC3728886; doi:10.7717/peerj.111)

A

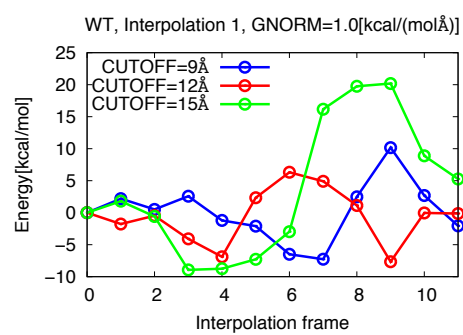

B

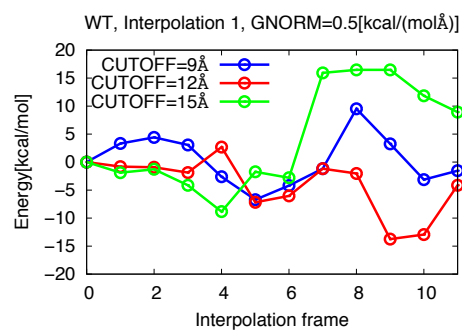

Supplement: Fig. S1 — εr = 78, CUTOFF referring to value used in geometry optimization.All SPE calculations done using CUTOFF = 15 Å. A: GNORM = 1.0 kcal/(mol Å). B: GNORM = 0.5 kcal/(mol Å). [file peerj-01-111-s002.pdf]

A

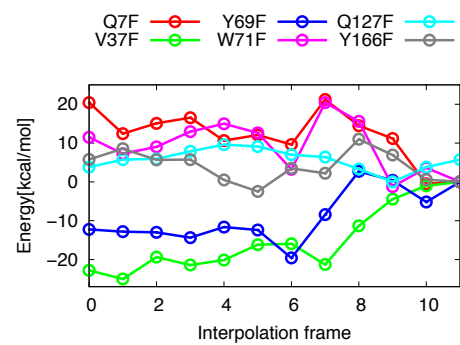

B

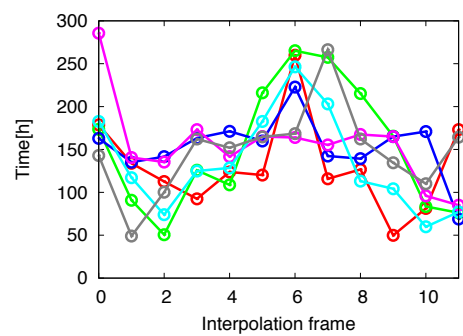

Supplement: Fig. S2 — In optimizations GNORM = 0.5 kcal/(mol Å), CUTOFF = 12 Å.In SPE calculations CUTOFF = 15 Å.A: Reaction barriers.B: Time requirements. [file peerj-01-111-s003.pdf]

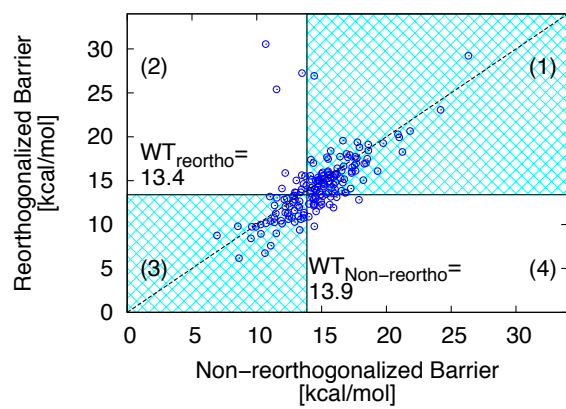

Supplement: Fig. S3 — Dependence of reaction barriers of single mutants on reorthogonalization.Independently of reorthogonalization, activity is qualitatively predicted the same for the data points within the highlighted areas.The number of data points in each quadrant are 90 (1), 14 (2), 45 (3) and 20 (4), quadrants indicated by “(i)” labels.Only data points lower than 34 kcal/mol shown. [file peerj-01-111-s004.pdf]
