# Supplementary material for: A computational method for the systematic screening of reaction barriers in enzymes: searching for Bacillus circulans xylanase mutants with greater activity towards a synthetic substrate
Source: PeerJ. 2013 Jul 23;1:e111. doi: 10.7717/peerj.111 (PMC3728886; doi:10.7717/peerj.111)

Position 7

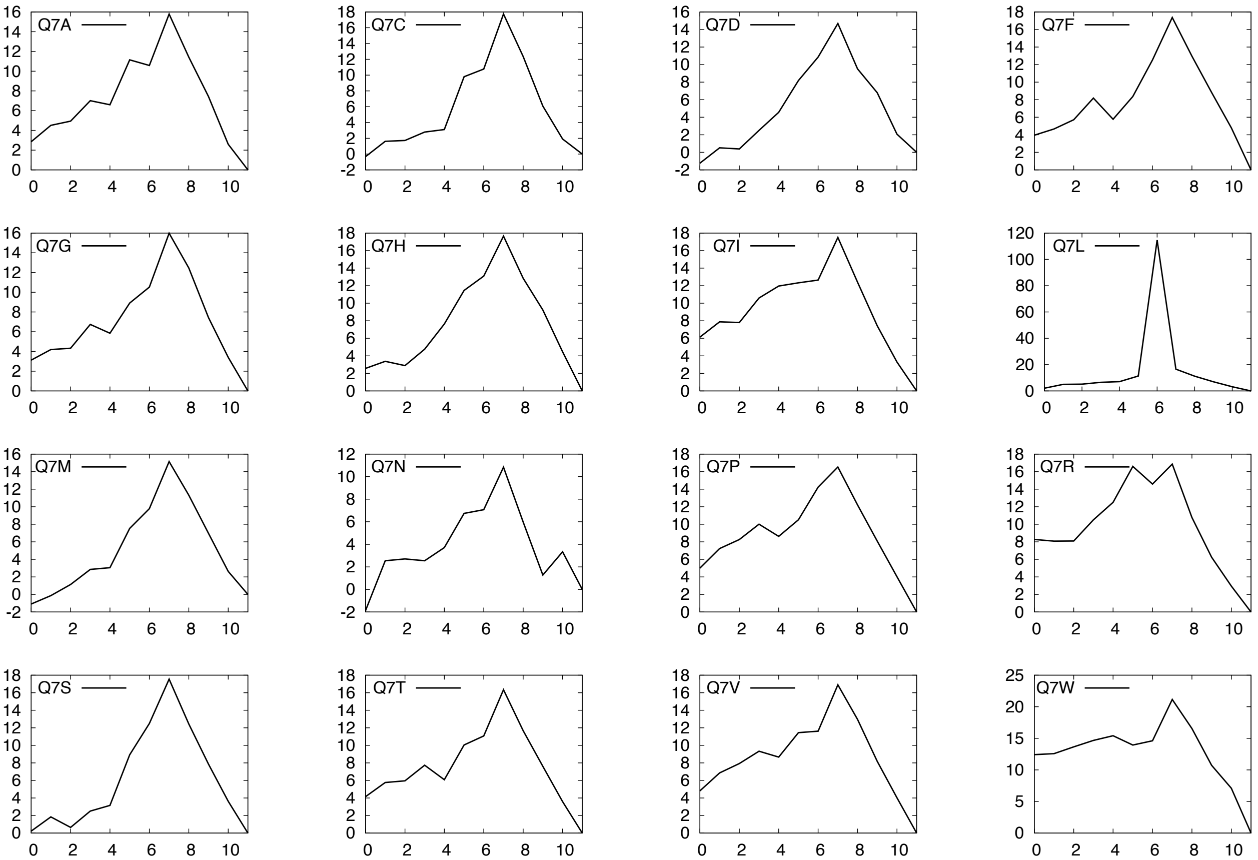

Position 9

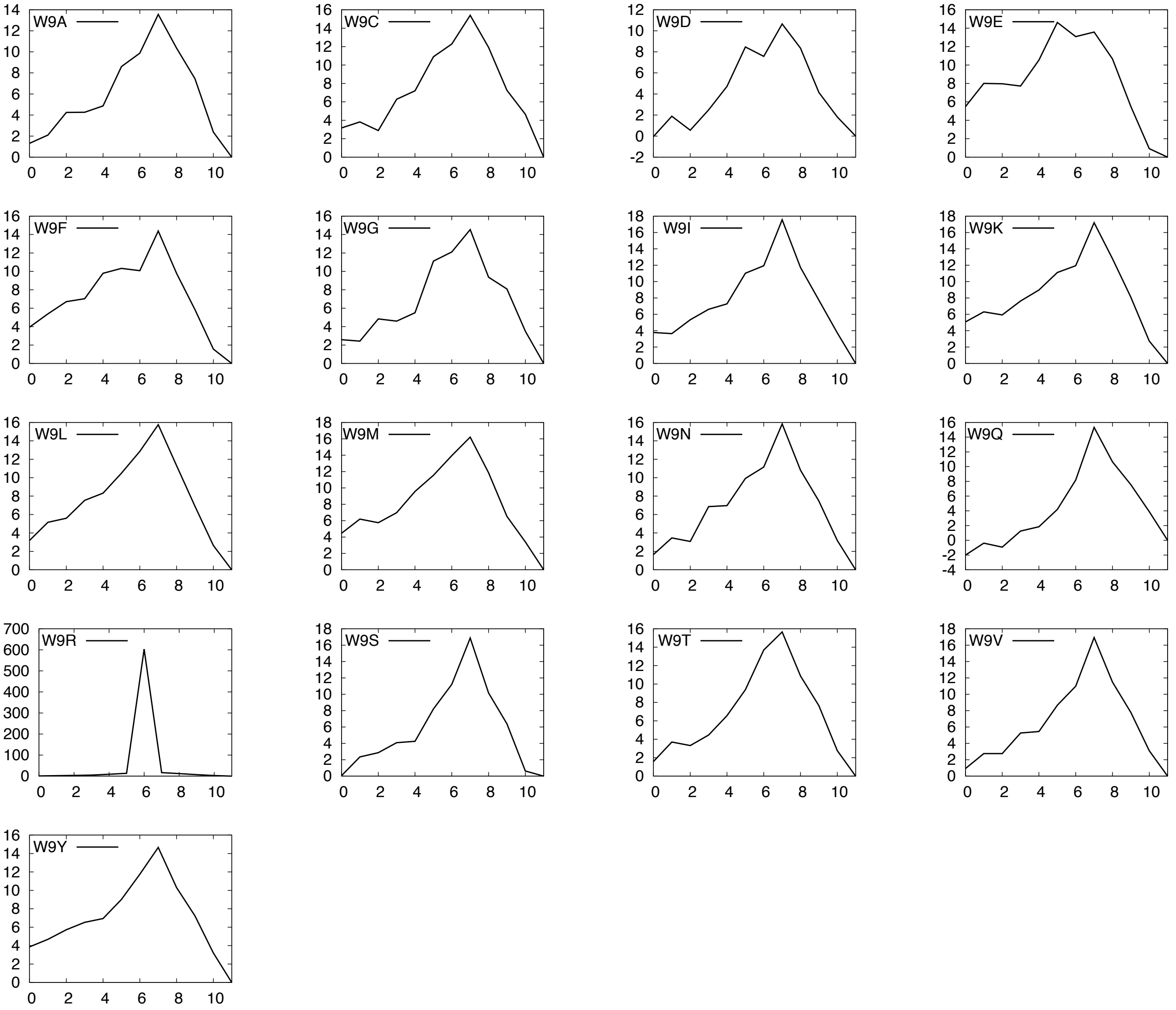
W9A
W9C
W9D
W9E
W9F
W9G
W9I
W9K
W9L
W9M
W9N
W9Q
W9R
W9S
W9T
W9V
W9Y

Position 35

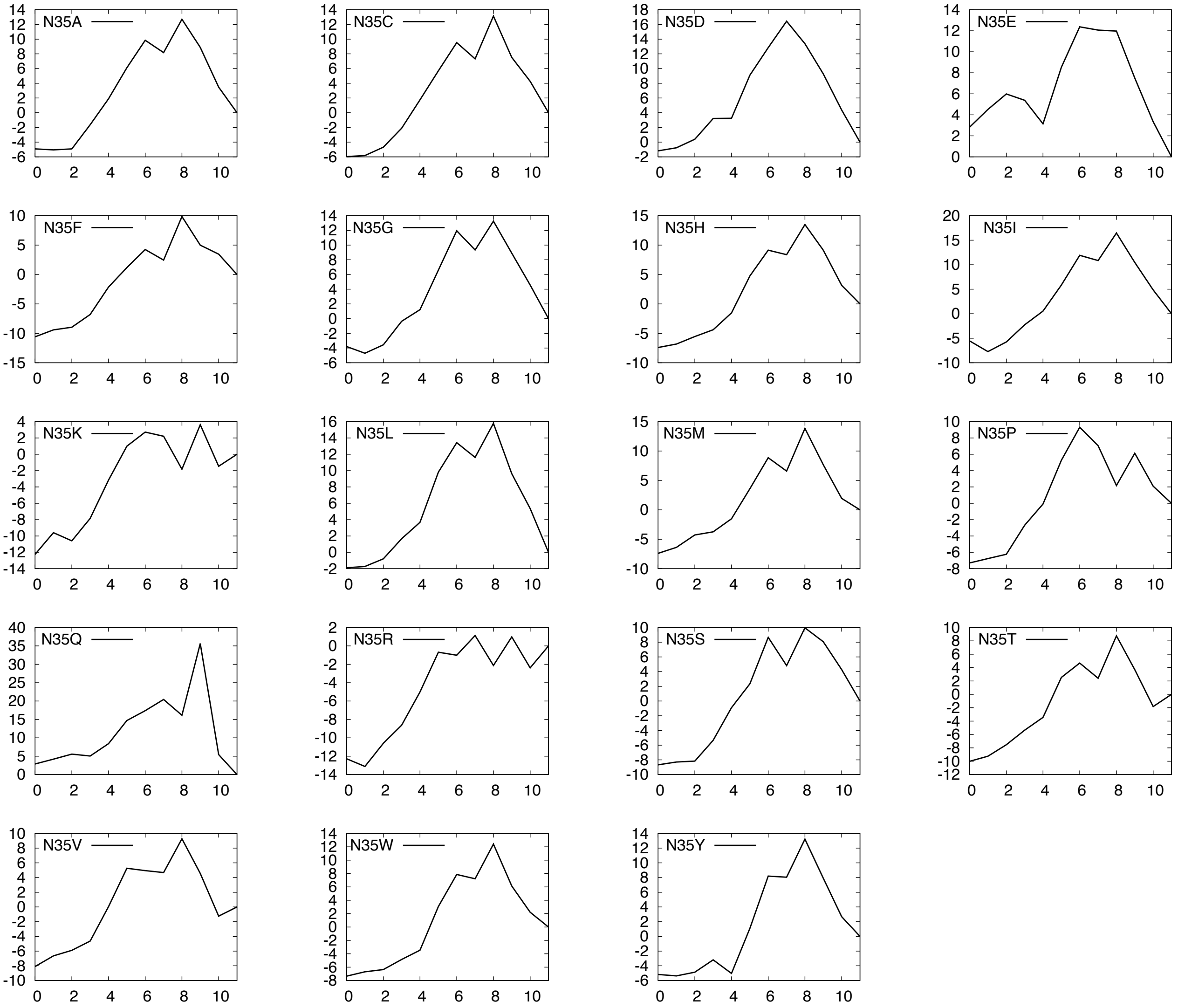
N35A
N35C
N35D
N35E
N35F
N35G
N35H
N35I
N35K
N35L
N35M
N35P
N35Q
N35R
N35S
N35T
N35V
N35W
N35Y

Position 37

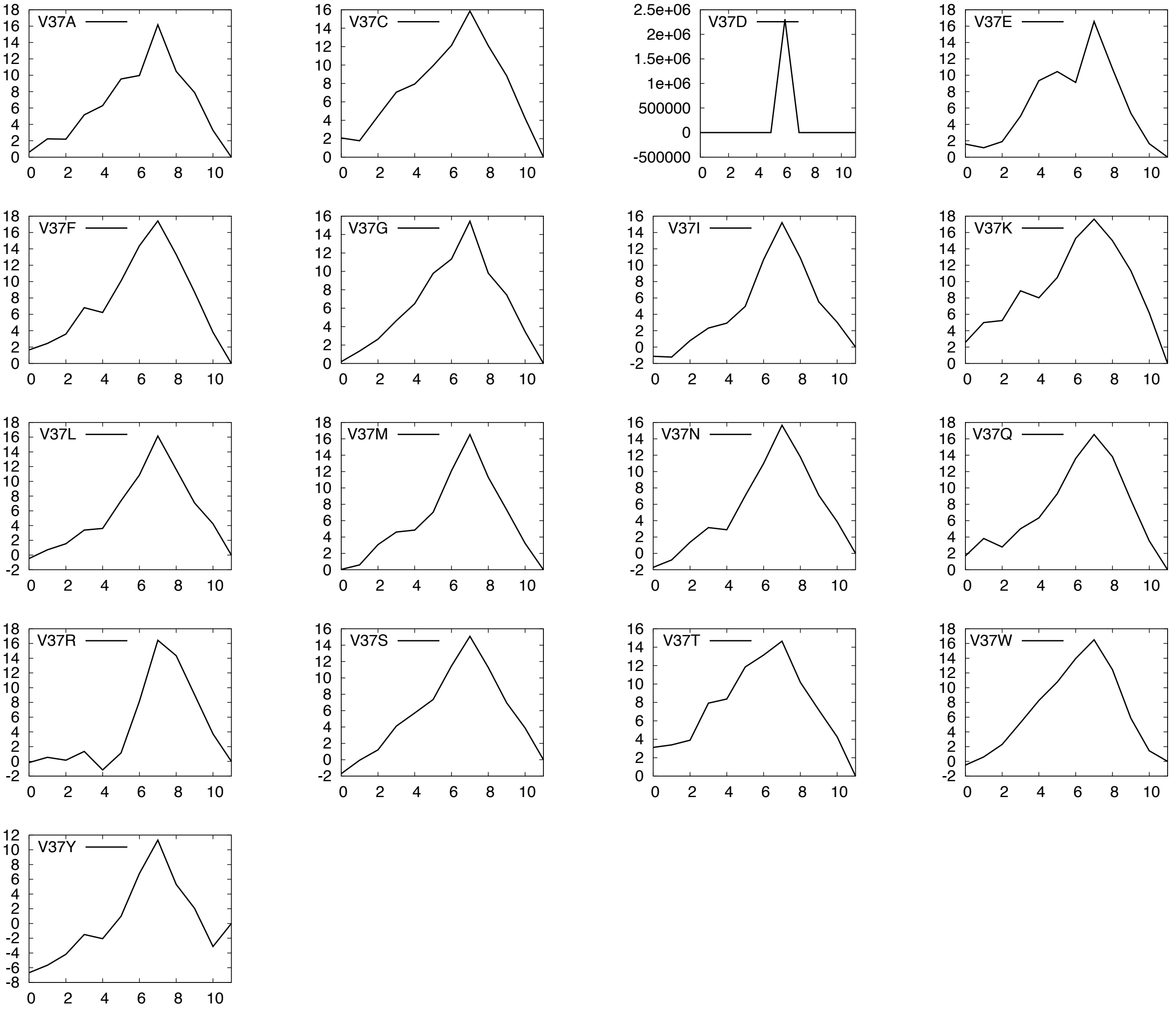

V37A
V37C
V37D
V37E
V37F
V37G
V37I
V37K
V37L
V37M
V37N
V37Q
V37R
V37S
V37T
V37W
V37Y

Position 65

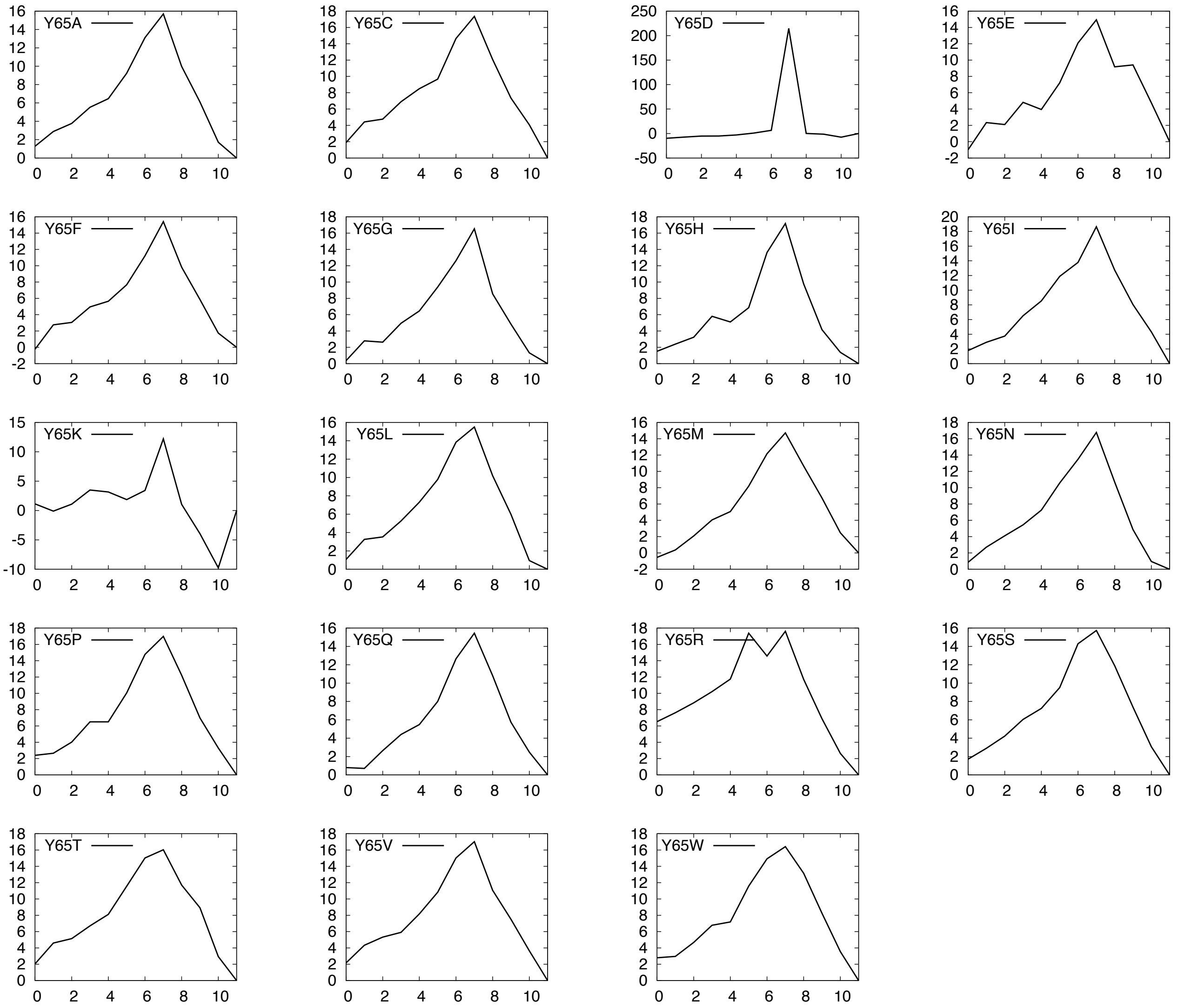
Y65A
Y65C
Y65D
Y65E
Y65F
Y65G
Y65H
Y65I
Y65K
Y65L
Y65M
Y65N
Y65P
Y65Q
Y65R
Y65S
Y65T
Y65V
Y65W

Position 69

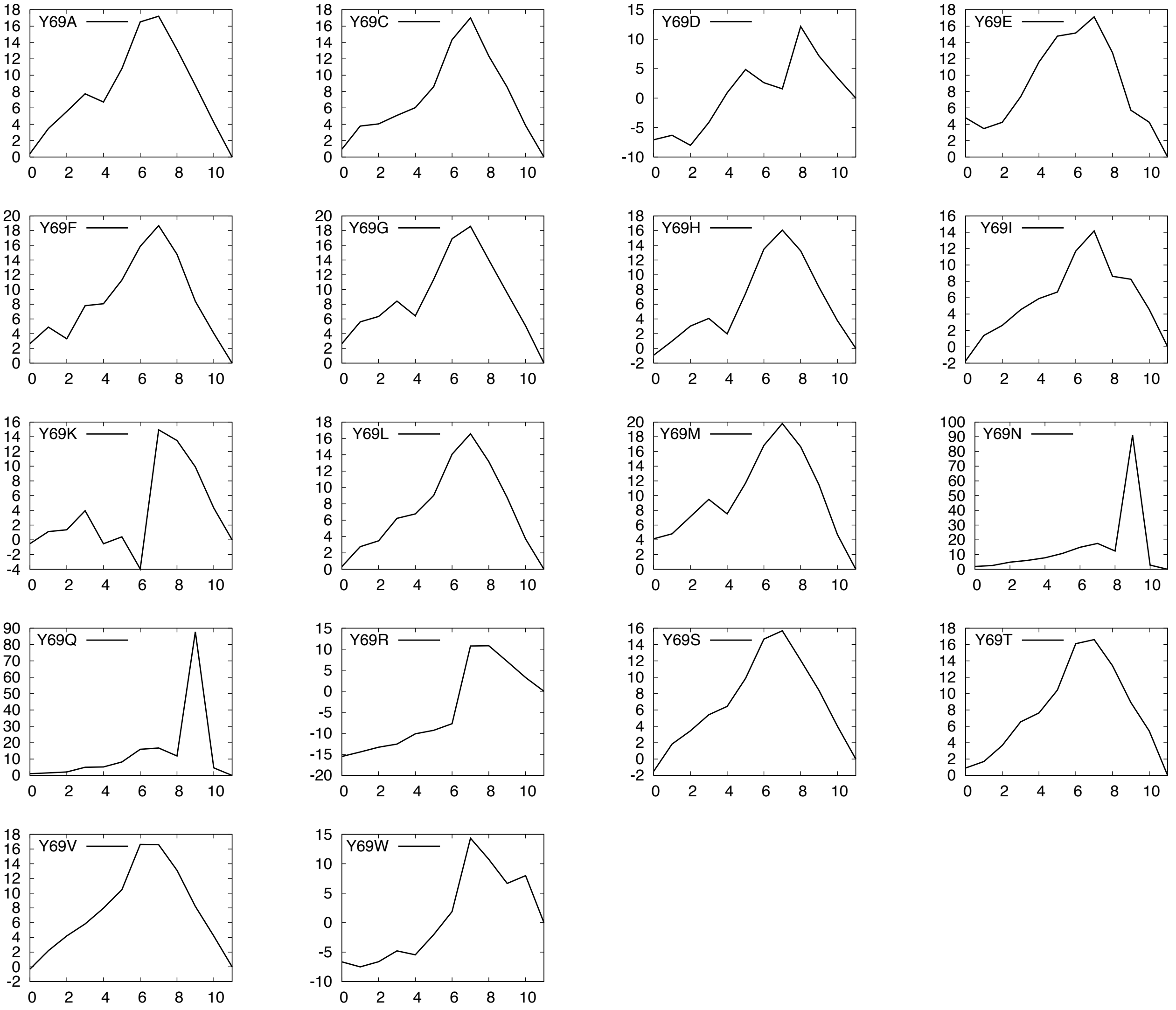
Y69A
Y69C
Y69D
Y69E
Y69F
Y69G
Y69H
Y69I
Y69K
Y69L
Y69M
Y69N
Y69Q
Y69R
Y69S
Y69T
Y69V
Y69W

Position 71

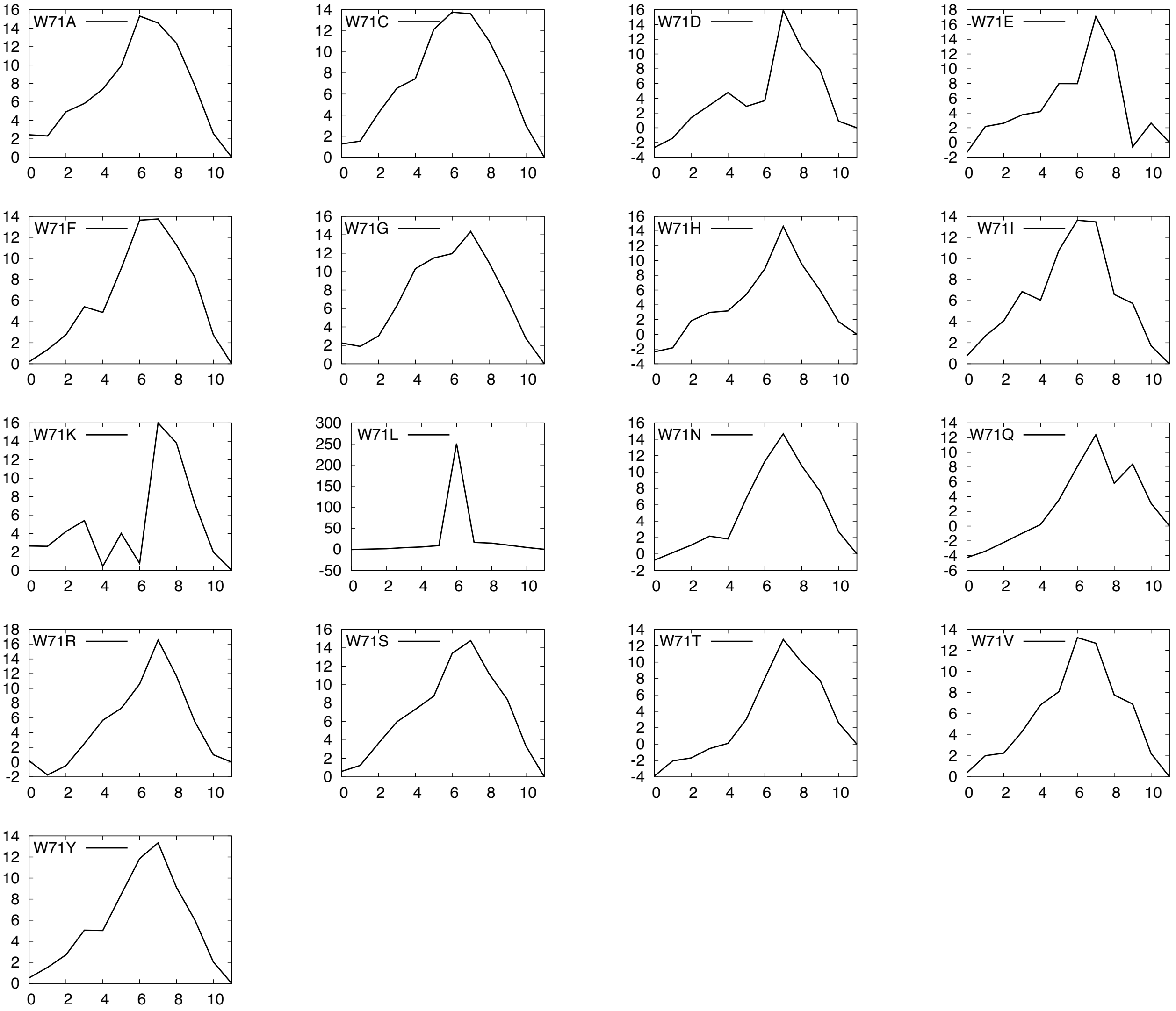

W71A
W71C
W71D
W71E
W71F
W71G
W71H
W71I
W71K
W71L
W71N
W71Q
W71R
W71S
W71T
W71V
W71Y

Position 80

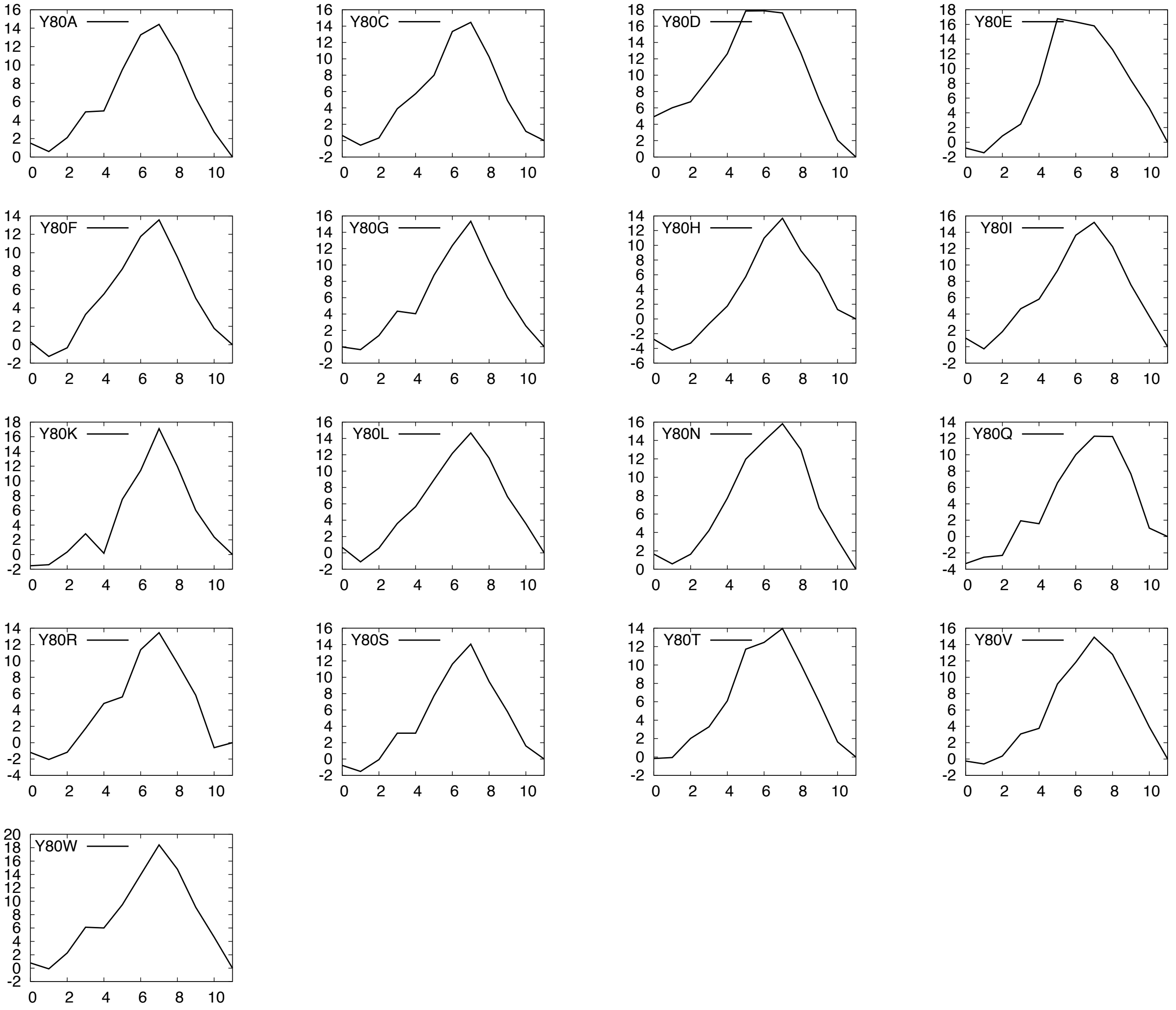
Y80A
Y80C
Y80D
Y80E
Y80F
Y80G
Y80H
Y80I
Y80K
Y80L
Y80N
Y80Q
Y80R
Y80S
Y80T
Y80V
Y80W

Position 112

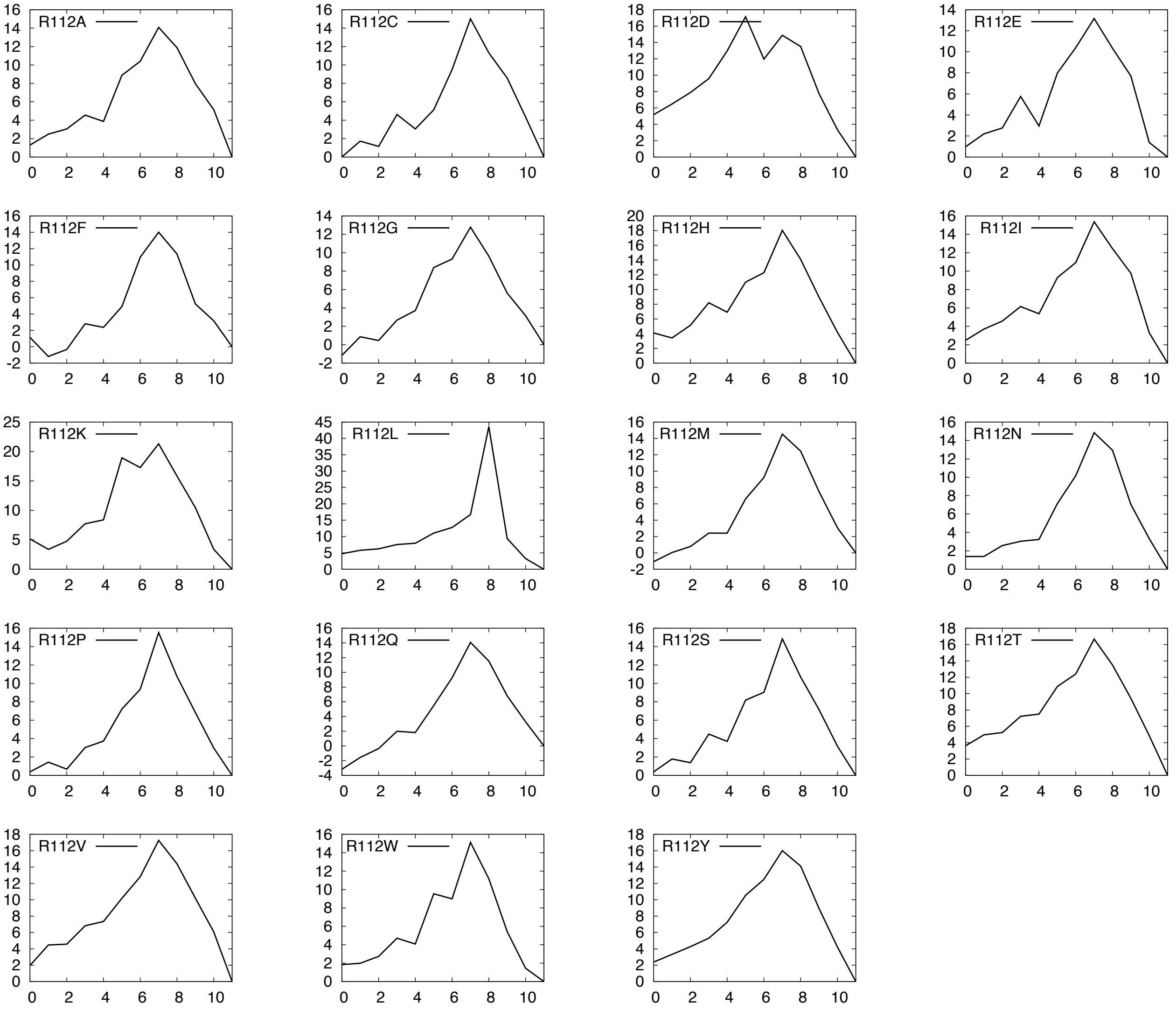

Position 115

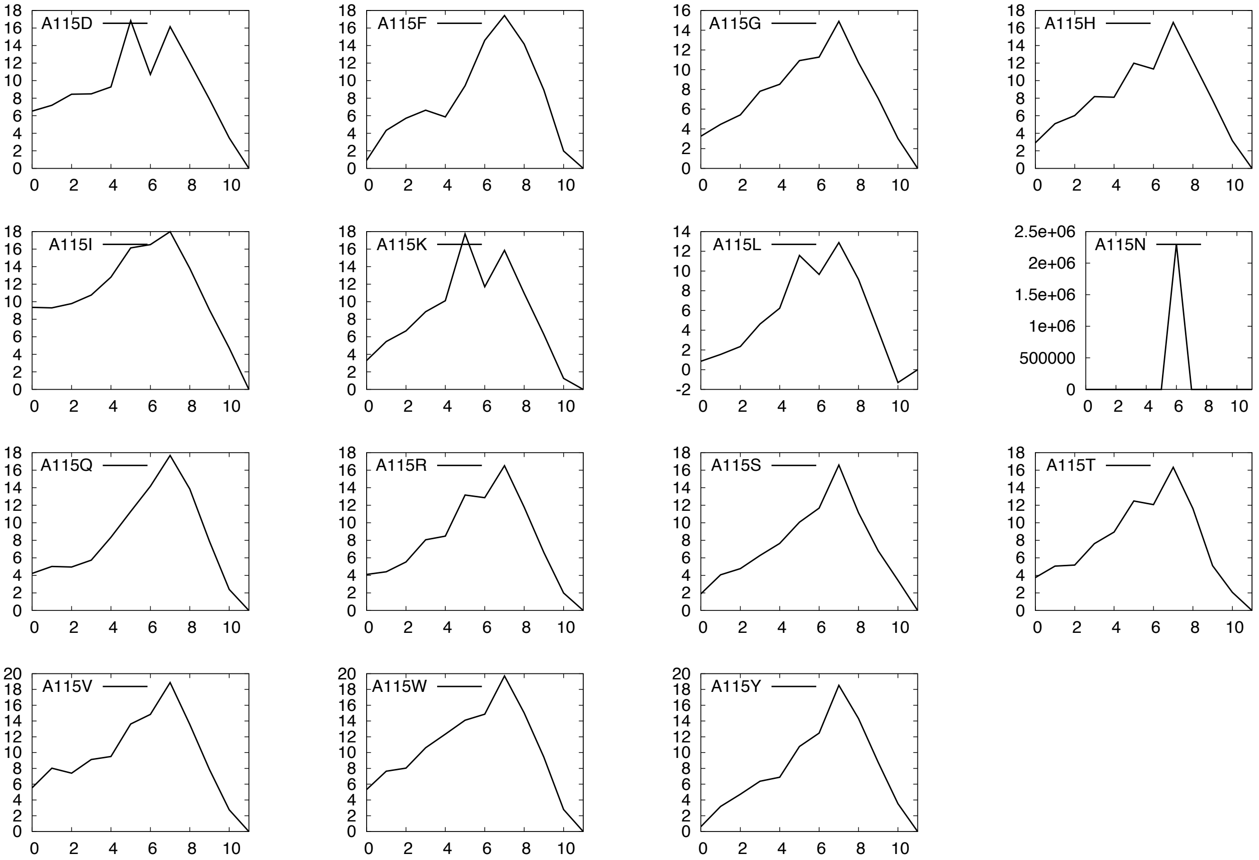

Position 116

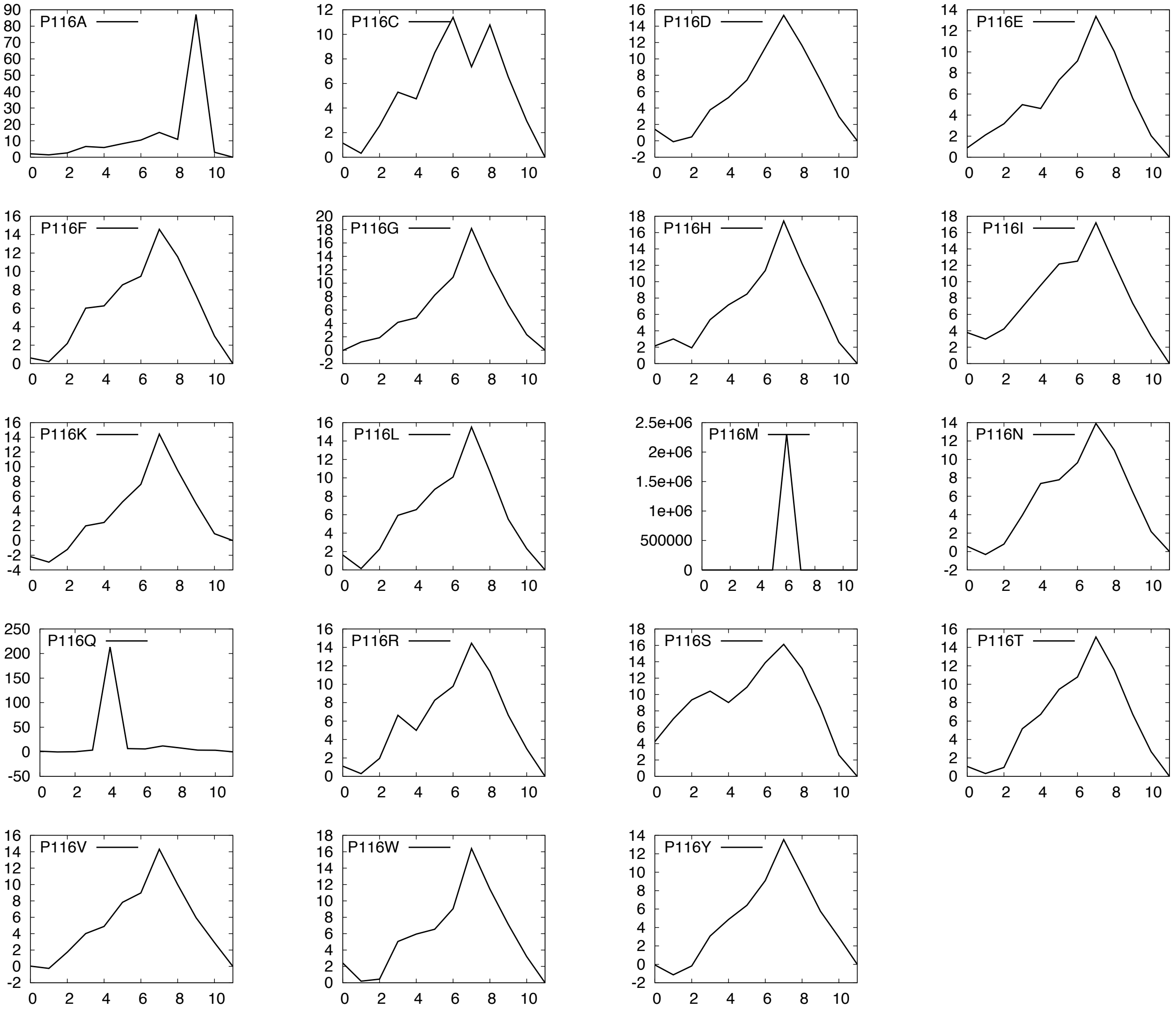

Position 117

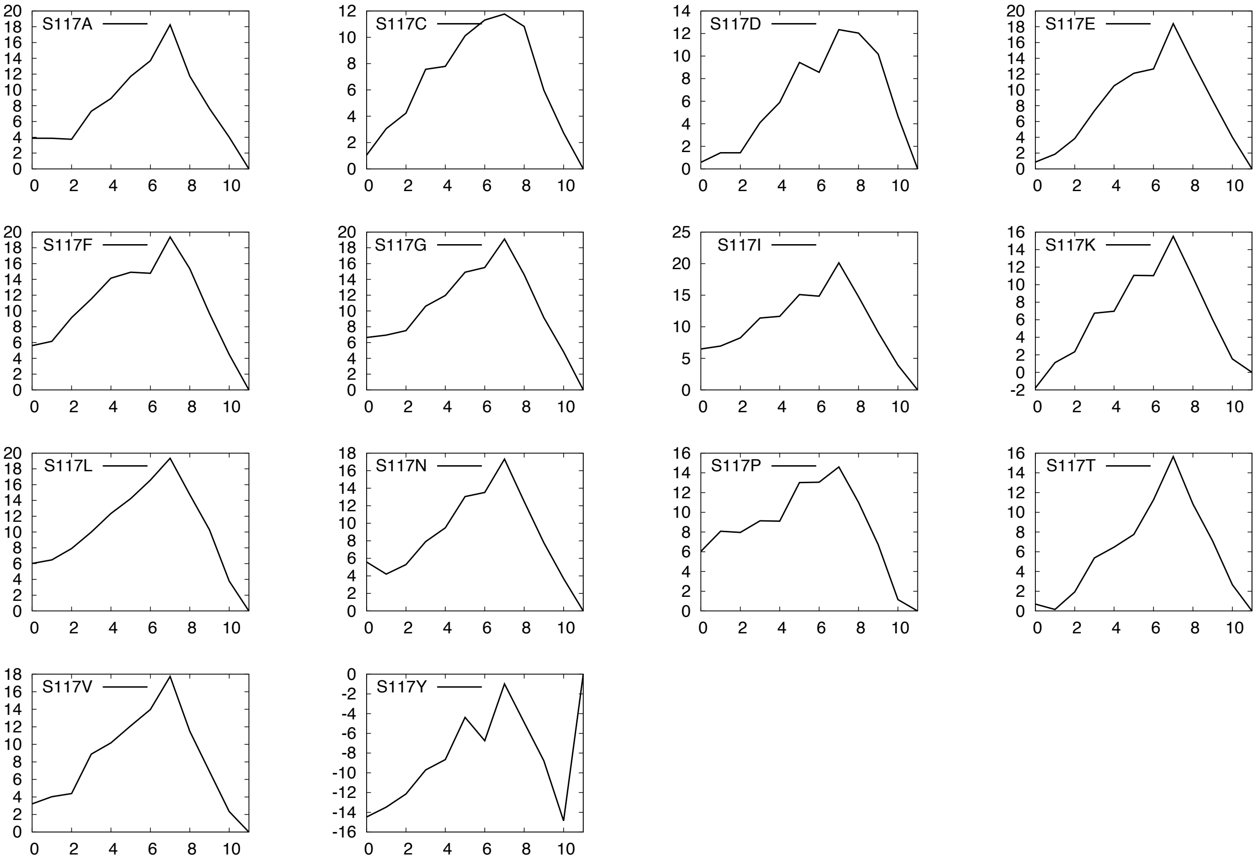

Position 118

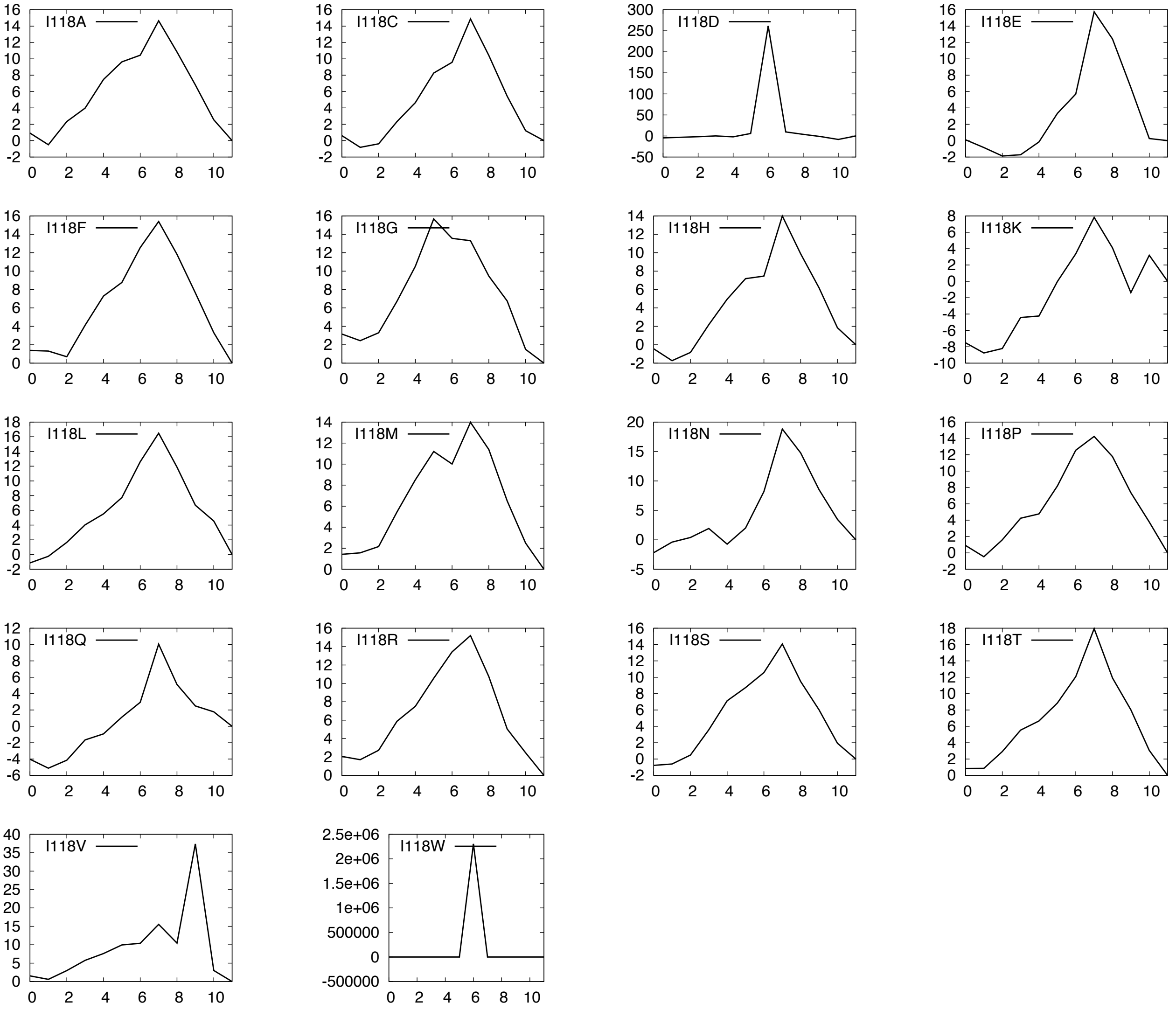

Position 125

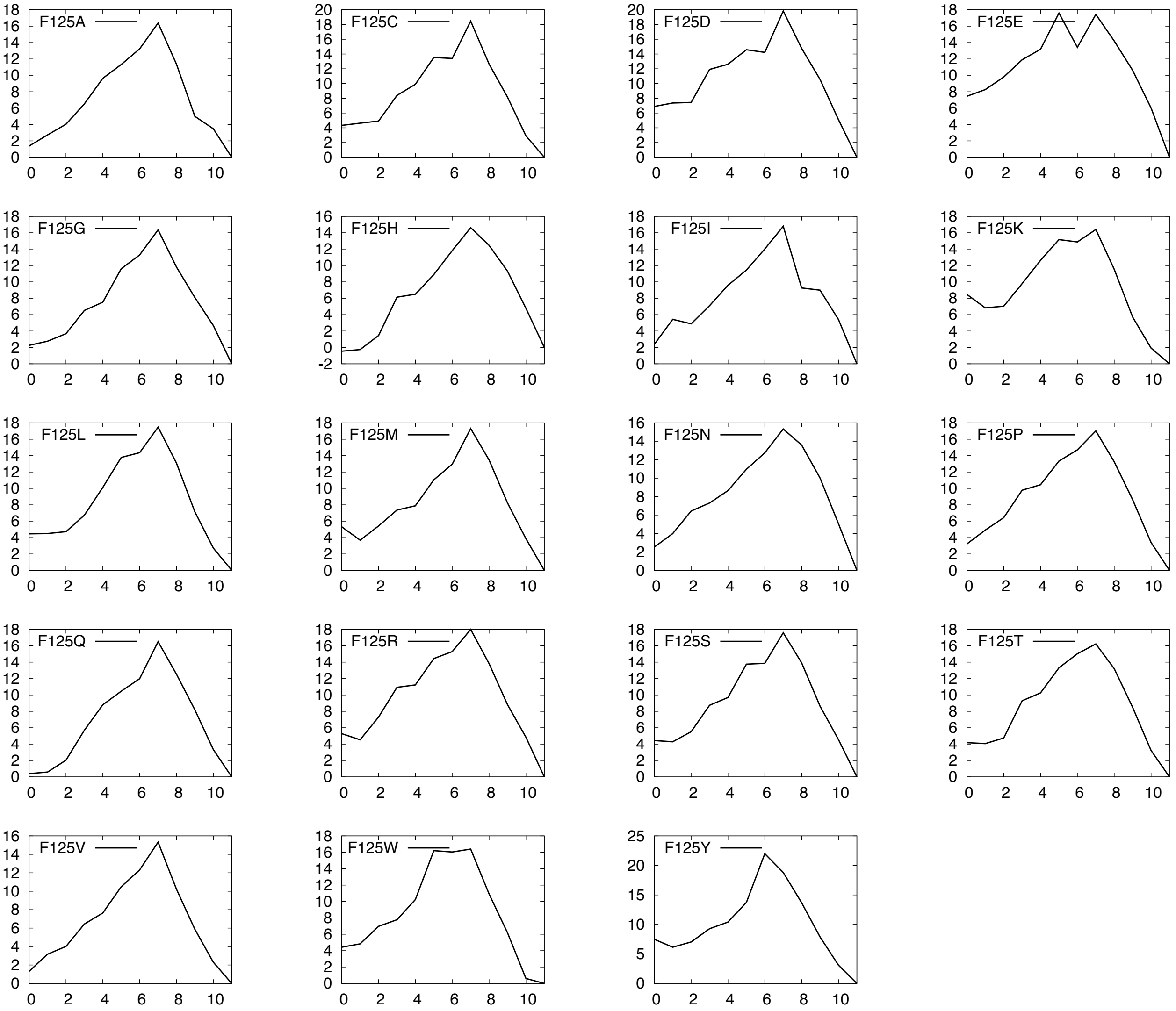

Position 127

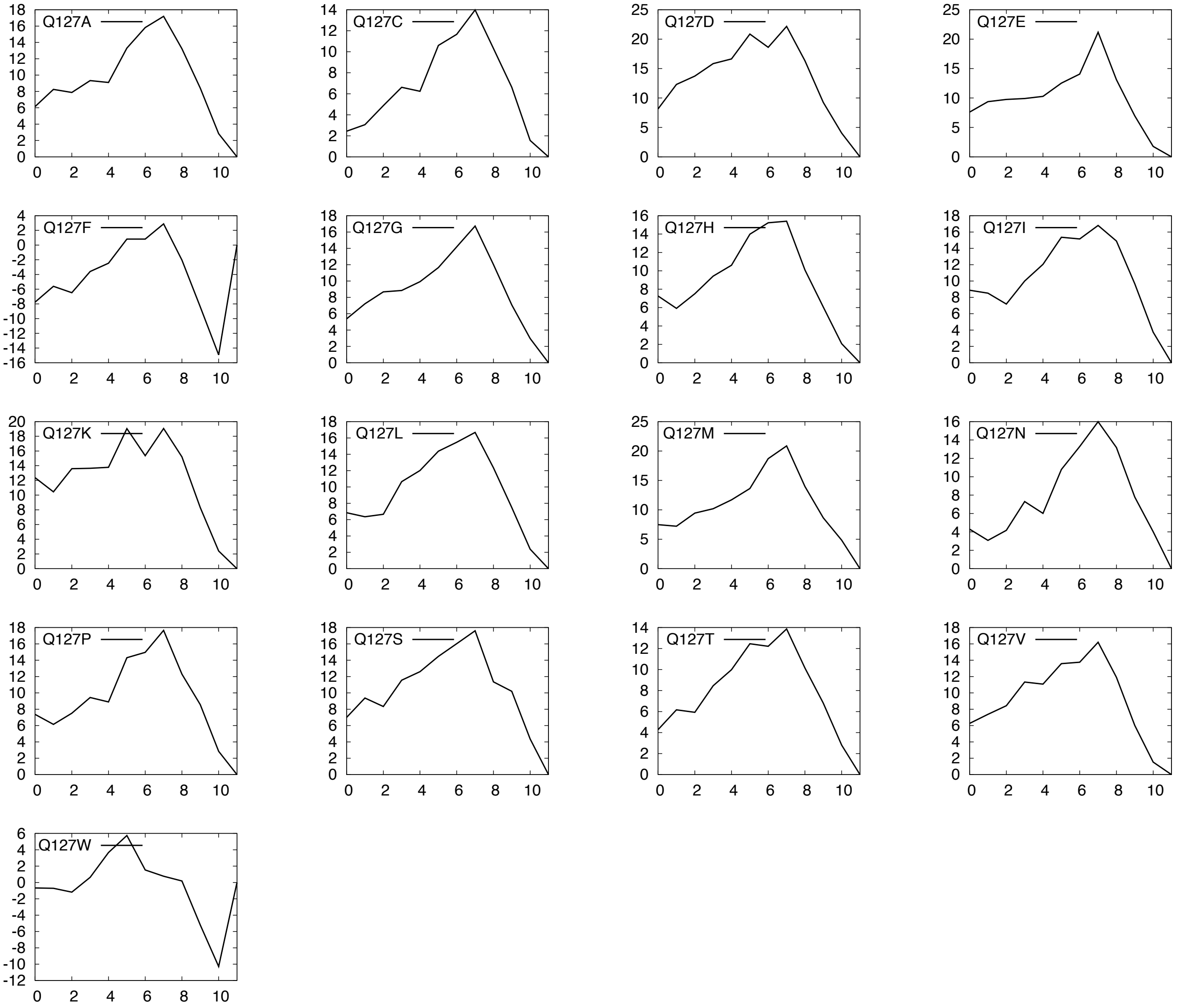

Position 129

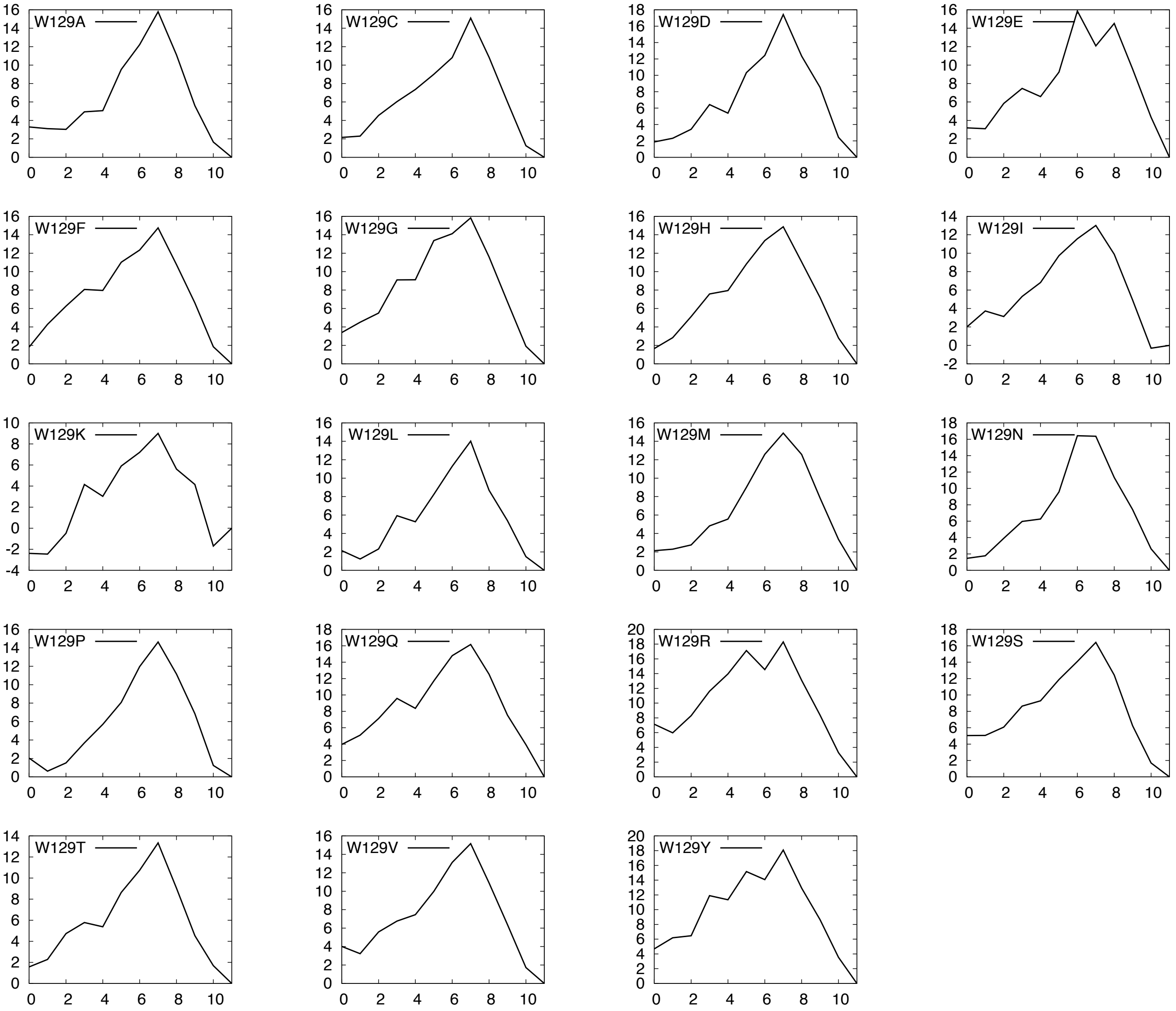

Position 166

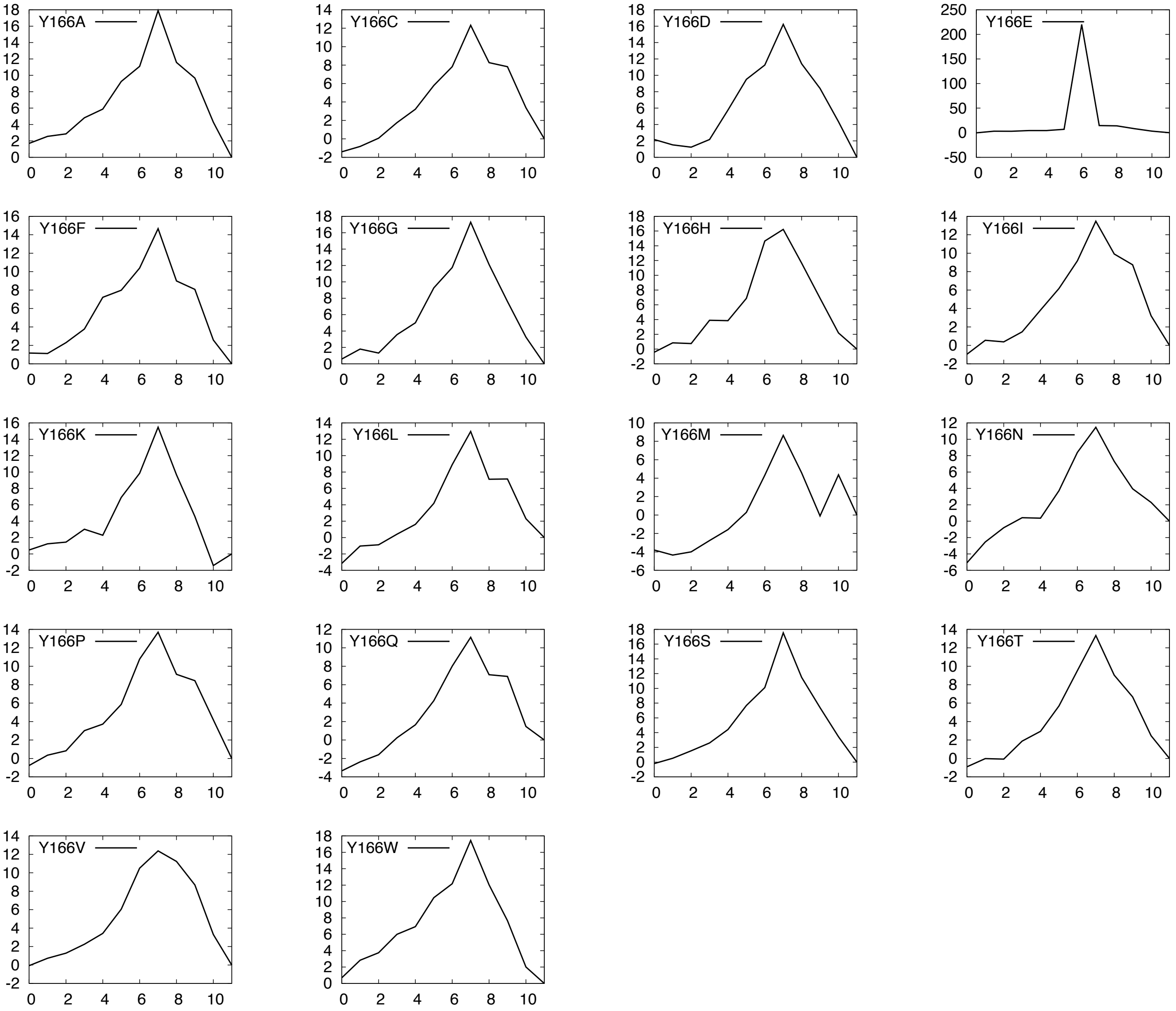

Position 174

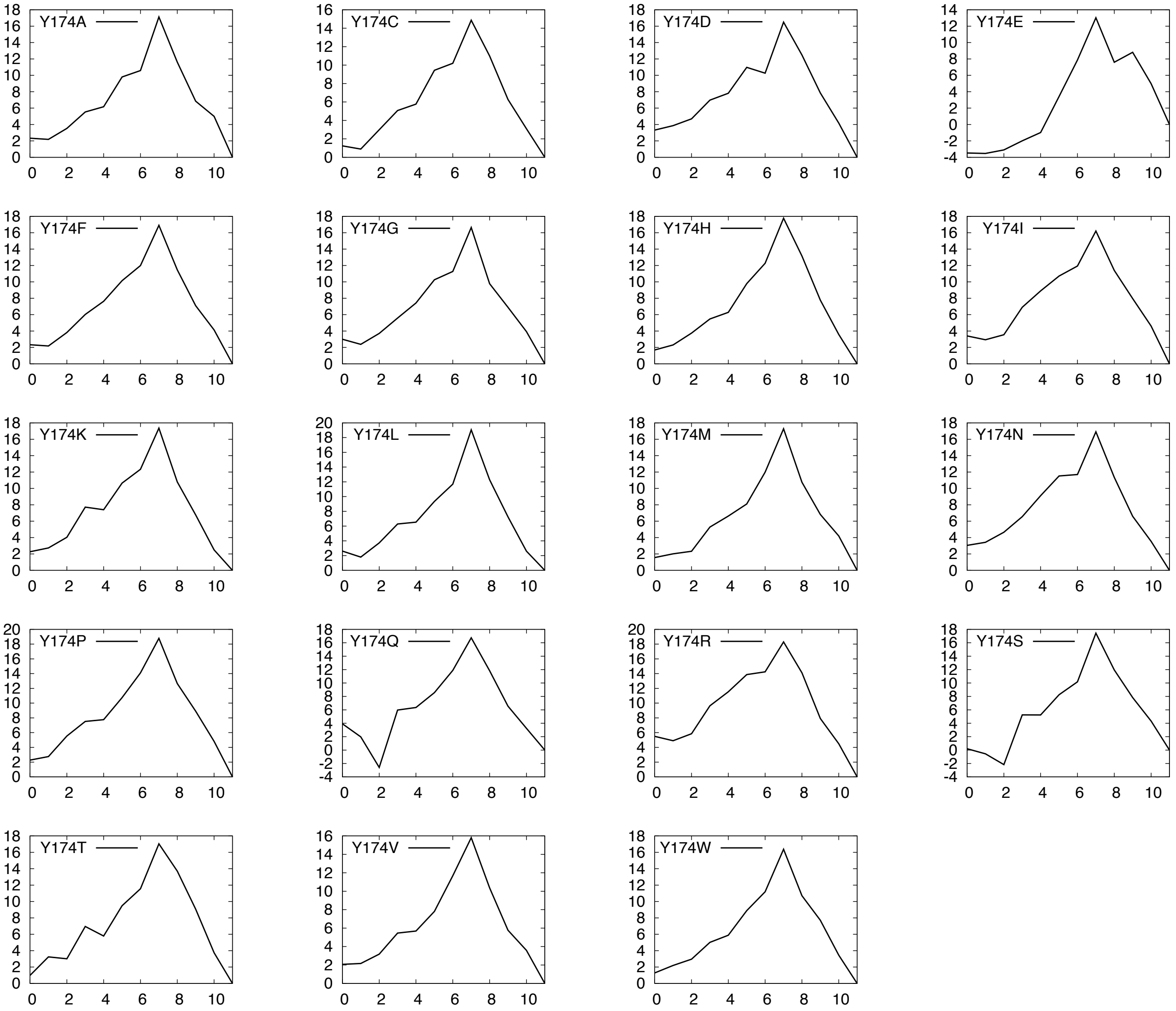

Supplement: Supplemental Information 5 [file peerj-01-111-s005.pdf]
