# Supplementary material for: A computational method for the systematic screening of reaction barriers in enzymes: searching for Bacillus circulans xylanase mutants with greater activity towards a synthetic substrate
Source: PeerJ. 2013 Jul 23;1:e111. doi: 10.7717/peerj.111 (PMC3728886; doi:10.7717/peerj.111)
Supplement: Table S1 [file peerj-01-111-s006.pdf]

Mutant	Reason for discarding
W9P	Local optimization of side chain gives unphysical structure
V37P	
Y69P	
Y80P	
W71P	
A115P	
I118Y	
Q7Y	Computed charge of GE structure not correct
V37H	
Y80M	
A115E	
A115M	
S117H	
S117M	
S117Q	
S117R	
S117W	
Q127Y	
Q7K	Computed charge of ES" wrong
A115C	
Q127R	
Q7E	Two atoms too close in automatically prepared structure
W9H	
W71M	
Y166R	
