# Supplementary material for: A computational method for the systematic screening of reaction barriers in enzymes: searching for Bacillus circulans xylanase mutants with greater activity towards a synthetic substrate
Source: PeerJ. 2013 Jul 23;1:e111. doi: 10.7717/peerj.111 (PMC3728886; doi:10.7717/peerj.111)
Supplement: Table S2 [file peerj-01-111-s007.pdf]

Mutant	Reason for discarding
Q7W-W9E	Geometry optimization time requirement too large
Q7W-P116C	
Q7W-F125K	
W9E-Y80D	
W9E-R112D	
W9E-W129I	
W9E-Y174D	
N35E-A115I	
V37T-Y65R	
V37T-Y69E	
V37T-I118M	
Y65R-R112D	
Y65R-F125K	
Y65R-Q127W	
Y69E-Y80D	
Y69E-Y174D	
W71G-Y127W	
W71G-R112D	
W71G-S117P	
W71G-W129I	
W71G-Y166V	
W71G-Y174D	
W71G-Y80D	
Y80D-Y116V	
R112D-F125K	
R112D-W129I	
R112D-Y166V	
A115I-P116C	
P116C-I118M	
S117P-F125K	
S117P-Y174D	
W129I-Y174D	
N35E-R112D	Computed charge of ES" wrong
N35E-W129I	
Y69E-S117P	
W71G-P116C	
S117P-Y166V	
Q7W-W71G	Two atoms too close in automatically prepared structure
Q7W-Y69E	
R112D-P116C	
R112D-Q127W	
R112D-S117P	
